# Changes in gene expression, cell physiology and toxicity of the harmful cyanobacterium *Microcystis aeruginosa* at elevated CO_2_

**DOI:** 10.3389/fmicb.2015.00401

**Published:** 2015-05-05

**Authors:** Giovanni Sandrini, Serena Cunsolo, J. Merijn Schuurmans, Hans C. P. Matthijs, Jef Huisman

**Affiliations:** ^1^Department of Aquatic Microbiology, Institute for Biodiversity and Ecosystem Dynamics, University of AmsterdamAmsterdam, Netherlands; ^2^Department of Aquatic Ecology, Netherlands Institute of EcologyWageningen, Netherlands

**Keywords:** bicarbonate uptake, climate change, CO_2_-concentrating mechanisms, harmful algal blooms, inorganic carbon uptake, microarrays, microcystins, phytoplankton

## Abstract

Rising CO_2_ concentrations may have large effects on aquatic microorganisms. In this study, we investigated how elevated pCO_2_ affects the harmful freshwater cyanobacterium *Microcystis aeruginosa*. This species is capable of producing dense blooms and hepatotoxins called microcystins. Strain PCC 7806 was cultured in chemostats that were shifted from low to high pCO_2_ conditions. This resulted in a transition from a C-limited to a light-limited steady state, with a ~2.7-fold increase of the cyanobacterial biomass and ~2.5-fold more microcystin per cell. Cells increased their chlorophyll *a* and phycocyanin content, and raised their PSI/PSII ratio at high pCO_2_. Surprisingly, cells had a lower dry weight and contained less carbohydrates, which might be an adaptation to improve the buoyancy of *Microcystis* when light becomes more limiting at high pCO_2_. Only 234 of the 4691 genes responded to elevated pCO_2_. For instance, expression of the carboxysome, RuBisCO, photosystem and C metabolism genes did not change significantly, and only a few N assimilation genes were expressed differently. The lack of large-scale changes in the transcriptome could suit a buoyant species that lives in eutrophic lakes with strong CO_2_ fluctuations very well. However, we found major responses in inorganic carbon uptake. At low pCO_2_, cells were mainly dependent on bicarbonate uptake, whereas at high pCO_2_ gene expression of the bicarbonate uptake systems was down-regulated and cells shifted to CO_2_ and low-affinity bicarbonate uptake. These results show that the need for high-affinity bicarbonate uptake systems ceases at elevated CO_2_. Moreover, the combination of an increased cyanobacterial abundance, improved buoyancy, and higher toxin content per cell indicates that rising atmospheric CO_2_ levels may increase the problems associated with the harmful cyanobacterium *Microcystis* in eutrophic lakes.

## Introduction

Cyanobacterial blooms are a well-known cause of nuisance in eutrophic lakes (Chorus and Bartram, 1999; Huisman et al., 2005; Merel et al., 2013). They may increase the turbidity of the water column, suppressing the growth of submerged water plants (Scheffer, 1998), and can produce considerable odor and taste problems (Watson et al., 2008). Moreover, cyanobacteria can produce a variety of potent toxins, including microcystins and anatoxins, which cause liver, digestive, and neurological disease in birds, mammals, and humans (Jochimsen et al., 1998; Carmichael, 2001; Codd et al., 2005). This has led to the closure of lakes and reservoirs for recreational use, aquaculture, drinking water, and irrigation water, often with considerable economic damage as a result (Verspagen et al., 2006; Dodds et al., 2008; Qin et al., 2010).

Several studies warn of an intensification of cyanobacterial blooms in eutrophic waters, due to the combination of global warming (Jöhnk et al., 2008; Paerl and Huisman, 2008; O'Neil et al., 2012) and rising CO_2_ concentrations in the atmosphere (Verschoor et al., 2013; Verspagen et al., 2014a,b). The dissolved inorganic carbon (C_i_) concentration in eutrophic lakes can vary strongly. Many lakes are supersaturated with dissolved CO_2_ (dCO_2_) with concentrations that can go up to 10,000 ppm (Cole et al., 1994; Sobek et al., 2005; Lazzarino et al., 2009), but lakes with high photosynthetic activities of dense cyanobacterial blooms have often undersaturating dCO_2_ concentrations (Ibelings and Maberly, 1998; Balmer and Downing, 2011; Verspagen et al., 2014b). Depletion of dCO_2_ during cyanobacterial blooms is accompanied by a strong increase in pH (Talling, 1976; Jeppesen et al., 1990; López-Archilla et al., 2004). With increasing pH, the speciation of dissolved inorganic carbon shifts from dCO_2_ toward bicarbonate (HCO^−^_3_) and carbonate (CO^2−^_3_). Because phytoplankton species differ in their uptake rates of dCO_2_ and bicarbonate (Rost et al., 2003; Price et al., 2008; Maberly et al., 2009), these changes in carbon speciation may strongly affect competitive interactions between the species (Tortell et al., 2002; Rost et al., 2008; Verschoor et al., 2013). In particular, cyanobacteria are believed to be very efficient competitors at low CO_2_ conditions (Shapiro, 1997).

Many cyanobacteria have evolved a highly efficient CO_2_-concentrating mechanism (CCM) to proliferate at low ambient dCO_2_ levels and to overcome the low affinity of the primary CO_2_ fixing enzyme, RuBisCO (Giordano et al., 2005; Price et al., 2008; Raven et al., 2012). The CCM of cyanobacteria works quite differently from CCMs found in eukaryotic algae. In the latter, a pH gradient across the chloroplast thylakoid membrane in the light secures C_i_ accumulation in the cytosol (Moroney and Ynalvez, 2007). In cyanobacteria, the CCM is based on accumulation of bicarbonate in specialized compartments, called carboxysomes, that contain the RuBisCO enzyme. Conversion of bicarbonate inside the carboxysomes by carbonic anhydrases raises the local CO_2_ concentration, near RuBisCO, which can then efficiently fix CO_2_ (Price et al., 2008). However, photorespiration and Mehler-like reactions have also been identified in cyanobacteria, signifying that cyanobacterial photosynthesis can still be limited by a low C_i_ availability despite their CCM (Helman et al., 2005; Zhang et al., 2009; Hackenberg et al., 2012). Up to four flavodiiron proteins (Flv1-4) are involved in the low C_i_ adaptation process, acting as an electron sink at photosystem I (PSI, for Flv1,3) and photosystem II (PSII, for Flv2,4) (Allahverdiyeva et al., 2011; Zhang et al., 2012; Bersanini et al., 2014).

The CCMs of fully sequenced cyanobacteria, like *Synechocystis* PCC 6803 and several *Synechococcus* strains, have been studied in detail (Omata et al., 1999; Price et al., 2004; Schwarz et al., 2011). This has revealed that cyanobacteria have several different C_i_ uptake systems: three systems for bicarbonate uptake have been identified and two systems for CO_2_ uptake (Price, 2011). BicA and SbtA are both sodium-dependent bicarbonate symporters. BicA has a low affinity and high flux rate, whereas SbtA has a high affinity and low flux rate (Price et al., 2004). The third bicarbonate uptake system, BCT1, has direct ATP-dependent functionality and consists of four subunits (CmpABCD). BCT1 has a relatively high affinity for bicarbonate and low flux rate, although its affinity is slightly lower than that of SbtA (Omata et al., 1999, 2002; Price et al., 2008). The two CO_2_ uptake systems, NDH-1_3_ and NDH1_4_, convert CO_2_ that passively diffuses into the cell to bicarbonate, and consist of multiple subunits (Price, 2011). The two CO_2_ uptake systems are likely NADPH-dependent (Price et al., 2002). Functionally, NDH-1_3_ has a high substrate affinity and low flux rate, whereas NDH-I_4_ combines a lower substrate affinity with a higher flux rate (Maeda et al., 2002; Price et al., 2002).

From an environmental and ecological perspective, the harmful cyanobacterium *Microcystis aeruginosa* (hereafter called *Microcystis*) is of great interest. *Microcystis* is one of the more notorious cyanobacterial species, producing dense and often toxic blooms in eutrophic lakes all over the world (Chen et al., 2003; Verspagen et al., 2006; Michalak et al., 2013). Now that full genome analysis has been completed for a range of different *Microcystis* strains (e.g., Kaneko et al., 2007; Frangeul et al., 2008; Humbert et al., 2013), knowledge on carbon acquisition is available, which enables detailed study of the response of *Microcystis* to rising CO_2_ levels. Selection experiments have shown that rising CO_2_ levels may shift the competitive balance between toxic and nontoxic *Microcystis* strains (Van de Waal et al., 2011). Furthermore, the genetic diversity of the CCM in different *Microcystis* strains was recently elucidated (Sandrini et al., 2014). This revealed that all investigated *Microcystis* strains contained genes encoding the two CO_2_ uptake systems, the bicarbonate uptake system BCT1 and several carbonic anhydrases, but the strains differ in the presence of the bicarbonate uptake genes *bicA* and *sbtA*.

In this study, we investigate the response of the toxic *Microcystis* strain PCC 7806 to rising CO_2_ levels, and how its gene expression and cell physiology, including the presence of its toxin microcystin, change at elevated pCO_2_. For this purpose, *Microcystis* was cultivated in controlled laboratory chemostats that were shifted from low to high pCO_2_ conditions. We provided the chemostats with a low CO_2_ partial pressure (pCO_2_) of 200 ppm to create C_i_-limited conditions. After 36 days, we raised the pCO_2_ level to 1450 ppm pCO_2_, which is a representative level for many supersaturated lakes (Sobek et al., 2005). Our results show that elevated CO_2_ leads to a highly specific transcriptome response of *Microcystis*, and affects the C_i_ uptake, biomass, cellular composition, and toxicity of *Microcystis* cells.

## Materials and methods

### Chemostat experiment

The axenic strain *Microcystis* strain PCC 7806 was obtained from the Pasteur Culture Collection (Paris, France). The strain was originally isolated from the Braakman reservoir, The Netherlands in 1972. It contains genes for the two CO_2_ uptake systems, the bicarbonate uptake systems BCT1 (high affinity) and BicA (low affinity), but lacks the high-affinity bicarbonate uptake system SbtA (Sandrini et al., 2014). The *Microcystis* strain was pre-cultured in four 2-L Erlenmeyer flasks using modified BG11 medium (Rippka et al., 1979) with 10 mmol L^−1^ NaNO_3_ and without Na_2_CO_3_ or NaHCO_3_, at an incident light intensity of 15 μmol photons m^−2^ s^−1^, 400 ppm CO_2_, and a temperature of 25°C. Regular microscopy did not reveal contaminations.

The experiment was carried out in four replicate chemostats designed for phytoplankton growth (Huisman et al., 2002) using the same modified BG11 medium. Each chemostat consisted of a flat culture vessel that was illuminated from one side (the front surface) to obtain a unidirectional light gradient. We applied a constant incident irradiance (*I_in_*) of 50 μmol photons m^−2^ s^−1^ using white fluorescent tubes (Philips Master TL-D 90 De Luxe 18 W/965, Philips Lighting, Eindhoven, The Netherlands). The chemostats had an optical path length of 5 cm and an effective working volume of 1.8 L. The temperature in the chemostats was maintained at 25°C using a stainless steel cooling finger connected to a water bath (Haake A28F/AC200; Thermo Fisher Scientific, Pittsburgh, PA, USA). The dilution rate was set at 0.01 h^−1^.

The chemostats were bubbled with CO_2_-enriched air at a flow rate of 25 L h^−1^. The CO_2_-enriched air was based on pressurized air (21% O_2_, 78% N_2_), from which the CO_2_ was removed by a CO_2_ adsorption air dryer (Ecodry K-MT6; Parker Zander, Lancaster, NY, USA) and four 1 m high columns filled with NaOH pellets. Subsequently, different amounts of pure CO_2_ gas were added using GT 1355R-2-15-A 316 SS Flow Controllers and 5850S Mass Flow Controllers (Brooks Instrument, Hatfield, PA, USA) to obtain the desired CO_2_ concentration. The gas mixture was moistened with 25°C water to suppress evaporation and led through a 0.20 μm Midisart 2000 filter (Sartorius Stedim Biotech GmbH, Göttingen, Germany) to sterilize the air, before it entered the chemostats. The CO_2_ concentration in the gas mixture was checked regularly with an Environmental Gas Monitor for CO_2_ (EGM-4; PP Systems, Amesbury, MA, USA).

Prior to the experiment, the four chemostats were inoculated with *Microcystis* from exponentially growing pre-cultures. The cultures were allowed to acclimate to the new chemostat conditions for 10 days. Subsequently, the cultures were diluted to an initial cyanobacterial abundance of ~60 mm^3^ L^−1^ to start the experiment. The CO_2_ concentration in the gas mixture was 200 ± 10 ppm CO_2_ during the first 36 days of the experiment (and also during the preceding acclimation phase). After 36 days, the CO_2_ concentration was raised to 1450 ± 50 ppm CO_2_. The experiment was run for 50 days, during which the chemostats were sampled 3–5 times per week.

### Light and cyanobacterial abundance

The incident irradiance (*I_in_*) and the irradiance penetrating through the chemostat vessel (*I_out_*) were measured with a LI-COR LI-250 quantum photometer (LI-COR Biosciences, Lincoln, NE, USA) at ten positions on the front and back surface of the chemostat vessels, respectively. Cell numbers, biovolumes and average cell size of samples were determined in triplicate using a Casy 1 TTC cell counter with a 60 μm capillary (Schärfe System GmbH, Reutlingen, Germany). Dry weight was determined in duplicate by filtering 0.75–10 mL of sample over pre-weighted 1.2 μm pore size 25 mm GF/C glass filters (Whatman GmbH, Dassel, Germany). Filters with cells were dried for 2 days in a 60°C oven, transferred to a desiccator to cool to room temperature, and subsequently weighted again.

### Aquatic chemistry

The pH of the samples was measured immediately after sample collection with a Lab 860 pH meter in combination with a BlueLine 28 Gel pH electrode (SCHOTT Instruments GmbH, Mainz, Germany). For dissolved inorganic carbon (DIC) concentration, alkalinity, and nitrate measurements, samples of 40 mL were immediately centrifuged (5 min at 4000 *g* and 20°C). The pellets were used for cellular C and N analysis, whereas the supernatant was filtered over 0.45 μm pore size 47 mm polyethersulfone membrane filters (Sartorius AG, Goettingen, Germany). The filtrate was transferred to sterile plastic urine tubes (VF-109SURI; Terumo Europe N.V., Leuven, Belgium). The tubes were filled completely and stored at 4°C until further analysis. DIC (three to five technical replicates) was measured with a TOC-V_CPH_ TOC analyzer (Shimadzu, Kyoto, Japan). Concentrations of dCO_2_, bicarbonate, and carbonate were calculated from DIC and pH (Stumm and Morgan, 1996). Alkalinity was determined with a 716 DMS Titrino titrator (Metrohm Applikon B.V., Schiedam, The Netherlands), based on the amount of 0.01 or 0.1 mol L^−1^ HCl added to reach pH 4.5 (ISO 9963-1:1994). The nitrate concentration was measured with a TNM-1 nitrogen detector (Shimadzu, Kyoto, Japan) attached to the TOC-V_CPH_ TOC analyzer.

### Cellular composition

Cellular C and N contents were determined by gently washing the pellets obtained from the previous centrifugation twice (5 min at 4000 *g* and 20°C) with a C- and N-free solution (10 mmol L^−1^ NaCl and 10 mmol L^−1^ K_2_HPO_4_ pH 8.0). The washed pellets were stored at −20°C until further analysis. Subsequently, the pellets were freeze dried, and the C and N content of 5 mg homogenized freeze-dried powder was analyzed using a Vario EL Elemental Analyzer (Elementar Analysensysteme GmbH, Hanau, Germany).

To determine the cellular carbohydrate, lipid, protein, and phosphate content, 2 mL samples were centrifuged and the pellets were stored at −20°C until further analysis. Carbohydrate was quantified using the phenol-sulphuric acid method, with D-glucose (Merck Millipore, Darmstadt, Germany) as standard (Dubois et al., 1956). Lipids were extracted with methanol and chloroform, and subsequently the lipid content was quantified using the sulphuric acid-vanillin-phosphoric acid method, with Triolein (>99%, Sigma-Aldrich Co, St Louis, MO, USA) as standard (Izard and Limberger, 2003). Proteins were quantified with the Bradford assay, using Brilliant Blue G-250 and bovine serum albumin as standard (Bradford, 1976). Cellular P was obtained by oxidizing cells with potassium persulfate for 1 h at 100°C (Wetzel and Likens, 2000), after which the phosphate concentration was measured colorimetrically according to Murphy and Riley (1962).

### *In vivo* absorption spectra

Light absorption spectra of the samples were scanned from 400 to 750 nm with a bandwidth of 0.4 nm using an Aminco DW-2000 double-beam spectrophotometer (Olis Inc., Bogart, GA, USA). BG11 medium without cells was used as baseline and the spectra were normalized based on the absorbance at 750 nm.

### Low temperature fluorescence

Relative amounts of PSI and PSII were determined using an Olis DM 45 77 K spectrofluorometer connected to an Olis photon counter (Olis Inc., Bogart, GA, USA). Glycerol (30% final concentration) was added to the samples, which were transferred to 3 mL polystyrene cuvettes (Sigma-Aldrich Co, St Louis, MO, USA) and frozen in liquid nitrogen. The excitation wavelength was 440 nm for chlorophyll *a*. Emission spectra of the samples were measured in triplicate at 600–750 nm, and normalized based on the mean emission at 600–660 nm.

### Microcystin analysis

*Microcystis* PCC 7806 produces two microcystin variants, [Asp^3^]MC-LR and MC-LR (Tonk et al., 2009). To determine the intracellular unbound microcystin content (cf. Meissner et al., 2013), 1–5 mL samples were filtered over 1.2 μm pore size 25 mm GF/C filters (Whatman GmbH, Dassel, Germany). The filters were stored at −20°C and subsequently freeze dried. Microcystins were extracted with 75% MeOH and analyzed with HPLC according to Van de Waal et al. (2011), using a Shimadzu LC-20AD HPLC system with a SPD-M20A photodiode array detector (Shimadzu, Kyoto, Japan). Peaks of [Asp^3^]MC-LR and MC-LR could not be completely separated. Therefore, both peaks were summed and are referred to as total microcystin. Earlier chemostat experiments showed that extracellular microcystin concentrations in the water phase were negligible (e.g., Van de Waal et al., 2011).

### RNA extraction

RNA samples for transcriptome analysis were taken from all four chemostats, at 0 h (just before increasing the pCO_2_) and at 0.75, 2, 4, 8, 24, 72, and 336 h after increasing the pCO_2_ from 200 to 1450 ppm. Fresh culture samples of ~40 mL were immediately cooled on ice and centrifuged for 5 min at 4000 *g* and 4°C in a pre-cooled centrifuge. The pellets were immediately resuspended in 1 mL TRIzol (Life Technologies, Grand Island, NY, USA), frozen in liquid nitrogen and stored at −80°C. Subsequently, RNA was extracted with TRIzol (Invitrogen) according to the supplier's instructions, using beads (0.5 mm BashingBeads; Zymo Research, Orange, CA, USA) to facilitate cell disruption. After the phase separation steps, the Direct-Zol™ RNA MiniPrep kit (Zymo Research) was used for RNA purification. The in-column DNase I digestion was included as well. RNA concentrations were quantified using a Nanodrop 1000 spectrophotometer (Thermo Scientific, San Jose, CA, USA), and all RNA samples had A_260_/A_280_ and A_260_/A_230_ values above 1.8. The quality of the RNA extracts was checked using an Agilent 2100 Bioanalyzer (Agilent Technologies, Amstelveen, the Netherlands).

### cDNA synthesis for microarray analysis

The cDNA library was prepared using the Superscript III Reverse Transcriptase kit (Invitrogen, Carlsbad, CA, USA) according to Eisenhut et al. (2007), with incorporation of Cy3-dUTP or Cy5-dUTP (Amersham Biosciences Benelux, Roosendaal, the Netherlands) in the cDNA. Non-incorporated fluorescent nucleotides were removed with the QIAquick PCR purification kit (Qiagen, Venlo, the Netherlands). The reverse transcription and fluorescent dye incorporation efficiency were monitored with a Nanodrop 1000 spectrophotometer (Thermo Scientific, San Jose, CA, USA).

### Microarray design

The transcriptome study was performed on two Agilent 8 × 60 K two-color microarray chips (Agilent Technologies, Amstelveen, the Netherlands). The microarray design and probe library of *Microcystis* PCC 7806 was based on Makower et al. (2015), which was partly derived from Straub et al. (2011). The sequence-specific oligonucleotide probes (60-mers) targeted 4691 protein-coding genes, which corresponds to 88.6% of the total predicted protein-coding genes in the annotated genome of *Microcystis* PCC 7806 (Frangeul et al., 2008). On average, five different probes per gene were used. Cy3- and Cy5-labeled cDNA probe samples from just before and at specific time points after increasing the pCO_2_ in the four chemostats were combined. A loop design was used, in which samples from the same chemostat but different time points were compared (Supplementary Table 1). The hybridization, washing and array reading were performed as described by Eisenhut et al. (2007).

### Microarray data analysis

The array data were analyzed with the R package Limma version 3.18.13 (Smyth, 2005; www.bioconductor.org; www.R-project.org). After background correction (“minimal method”), within-array normalization was applied using global loess normalization. Subsequently, the data was log2 transformed. Between-array normalization was applied using A-quantile normalization (Bolstad et al., 2003). Log2 expression values for the different time points (after increasing the pCO_2_) were calculated with respect to the reference value at *t* = 0 (just before increasing the pCO_2_). Expression values of multiple probes per gene were averaged. Hence, for each gene we obtained four expression values per time point, based on the four replicate chemostats. For each time point, *p*-values were calculated from the moderated *t*-statistic, and were adjusted for multiple hypothesis testing by controlling the false discovery rate (Storey and Tibshirani, 2003). Log2 gene expression values of < -0.9 or > 0.9 (similar to Nodop et al., 2008; Schwarz et al., 2011), and *p* < 0.01 were considered differentially expressed. Visual representation was done using hierarchical clustering and heatmaps with the hclust and heatmap.2 functions of the R gplots package version 2.13.0. The expression values, corresponding *p*-values and gene function according to CyanoBase (http://genome.microbedb.jp/cyanobase/Synechocystis/genes/category) of all investigated genes are listed in Supplementary Table 2.

### RT-qPCR gene expression analysis

To validate the microarray results, we also investigated changes in gene expression of several CCM genes and the *mcyB* gene using RT-qPCR. We designed gene-specific primers (Supplementary Table 3). Secondary structure analysis of the primers was checked with OligoAnalyzer 3.1 (Integrated DNA Technologies, Coralville, IA, USA) and the *in silico* specificity was assessed using Primer-BLAST (Ye et al., 2012). The specificity of the primers was confirmed with normal PCR on genomic DNA using the GoTaq® Hot Start DNA polymerase kit (Promega GmbH, Mannheim, Germany) combined with gel electrophoresis.

For the RT-qPCR gene expression analysis, we used the same RNA samples as for the microarray analysis at the time points 0, 4, and 336 h (2 weeks) after increasing the pCO_2_. cDNA was synthesized by reverse transcription of the RNA samples with Superscript III (Invitrogen) according to the supplier's instructions, using 250 ng random hexamers (Amersham Biosciences Benelux, Roosendaal, the Netherlands) and 5 μg of pure RNA in a total reaction volume of 20 μL. Next, the RNase H treatment (New England Biolabs, Ipswich, MA, USA) was applied to remove any RNA complementary to the cDNA. Subsequently, the qPCR Maxima® SYBR Green Master Mix (2x) (Thermo Fisher Scientific, Pittsburgh, PA, USA) was applied on the cDNA samples according to the supplier's instructions, in an ABI 7500 Real-Time PCR system (Applied Biosystems, Foster City, CA, USA). The two step cycling protocol was used, with a denaturation temperature of 95°C (15 s) and a combined annealing/extension temperature of 60°C (60 s) during 40 cycles. The reactions contained 0.3 μmol L^−1^ primers and 1 μL of 10 times diluted cDNA from the RT reaction in a total reaction volume of 25 μL. Other reaction components were added as instructed by the supplier. ROX solution was used to correct for any well-to-well variation and melting curve analysis was performed on all measured samples to rule out non-specific PCR products.

Amplification efficiencies of individual runs (E) were calculated with LinRegPCR (version 2012.3; Ramakers et al., 2003; Ruijter et al., 2009) and were between 1.8 and 2.0 (Supplementary Table 3). Time point 0 (just before the pCO_2_ was increased) was used as reference sample, and 16S rRNA was used as reference gene. Each RT-qPCR plate contained a reference sample and samples with primers targeting 16S rRNA to overcome plate effects. For each individual sample, two technical replicates were measured and the average of the two measurements was used in the data analysis. The data were baseline corrected using LinRegPCR, and the same software was used to calculate quantification cycle (C_q_) values. LinRegPCR did not detect samples without amplification, without a plateau, with a baseline error or noise error, or with deviating amplification efficiencies. Negative control samples did not show significant amplification. For each gene, we calculated the relative gene expression with respect to time point 0 using 16S rRNA as reference gene according to the 2^−ΔΔCt^ method (Livak and Schmittgen, 2001). MS Excel version 2010 and SPSS version 20.0 were used for the statistical analyses. A one-tailed *t*-test was used to assess whether the change in gene expression with respect to time point 0 was significant (again with *n* = 4 expression values per time point, based on the four replicate chemostats). An independent samples *t*-test was used to detect differences between the 4 h and 2 week samples. The false discovery rate (FDR) was used to correct for multiple hypothesis testing (Benjamini and Yekutieli, 2001; FDR adjusted *p* < 0.05).

### O_2_ optode experiments

In mineral medium without nitrate, the O_2_ evolution rate of cyanobacterial cells exposed to saturating light provides a good proxy of their C_i_ uptake rate (Miller et al., 1988). We therefore measured O_2_ evolution with an Oxy-4 mini O_2_ optode (PreSens GmbH, Regensburg, Germany) to study the C_i_ uptake of samples from the steady-state chemostats at 200 and 1450 ppm pCO_2_. External carbonic anhydrase activity in *Microcystis* was previously shown to be negligible (Song and Qiu, 2007). Fresh cell samples were pelleted (4000 *g* for 5 min at 20°C), washed 1x and then resuspended in C*_i_*- and nitrate-depleted modified BG11 medium (no added NaNO_3_ and NaCO_3_/NaHCO_3_, but with 25 mmol L^−1^ NaCl and 10 mmol L^−1^ CAPSO-NaOH added at pH 9.8; the medium was aerated with N_2_ gas for 4 h before usage). The absorbance at 750 nm (A_750_) of washed and resuspended samples was 0.300. Three mL of sample was inserted into custom-made glass incubation chambers (four measurements were done at the same time), each connected to an O_2_ optode. The glass chambers were connected to a RM6 water bath (Lauda, Lauda-Königshofen, Germany) to keep the temperature of the samples at 20.3°C. Magnetic stirring was used for mixing. The O_2_ optode sensors were calibrated with N_2_ gas (“0%”) and air (“100%”). A saturating amount of light (500 μmol photons m^−2^ s^−1^) was used, which was provided by KL1500 compact Schott lamps (Schott AG, Mainz, Germany). Subsequently, specific amounts of NaHCO_3_ were added (5, 20, 100, 300, 1000, or 10,000 μmol L^−1^), after which the rate of O_2_ evolution (μmol L^−1^ min^−1^) was measured. The evolution was expressed per mg of chlorophyll *a*. Photosynthetic pigments were extracted with 90% acetone and analyzed using HPLC with photodiode array detection (LC-20AD liquid chromatographs, SIL-20A auto sampler, CTO-20AC column oven, SPD-M20A diode array detector, RF-10A*_XL_* fluorescence detector; Shimadzu, Kyoto, Japan). Identification of chlorophyll *a* was based on the characteristic absorbance at 664 nm and the specific retention time compared with a standard obtained from DHI Group (Copenhagen, Denmark).

To evaluate the activity of the different C_i_ uptake systems, we investigated O_2_ evolution in the presence and absence of 25 mmol L^−1^ LiCl. Lithium ions are known to inhibit the activity of the sodium-dependent bicarbonate uptake systems (e.g., Espie et al., 1988). The experiments were performed at pH 7.8 (using 10 mmol L^−1^ TES-KOH pH 7.8 buffer) and pH 9.8 (using 10 mmol L^−1^ CAPSO-KOH pH 9.8 buffer) to vary the relative availability of CO_2_ and HCO^−^_3_, using C_i_ and N depleted modified BG11 medium containing 25 mmol L^−1^ NaCl to which 200 μmol L^−1^ KHCO_3_ was added after initial C_i_ depletion. To test if O_2_ evolution rates differed significantly between the treatments, one-way analysis of variance was used with *post-hoc* comparison of the means based on Tukey's test (*n* = 4 replicates per treatment).

## Results

### Population dynamics and inorganic carbon chemistry

**Figure 1:**
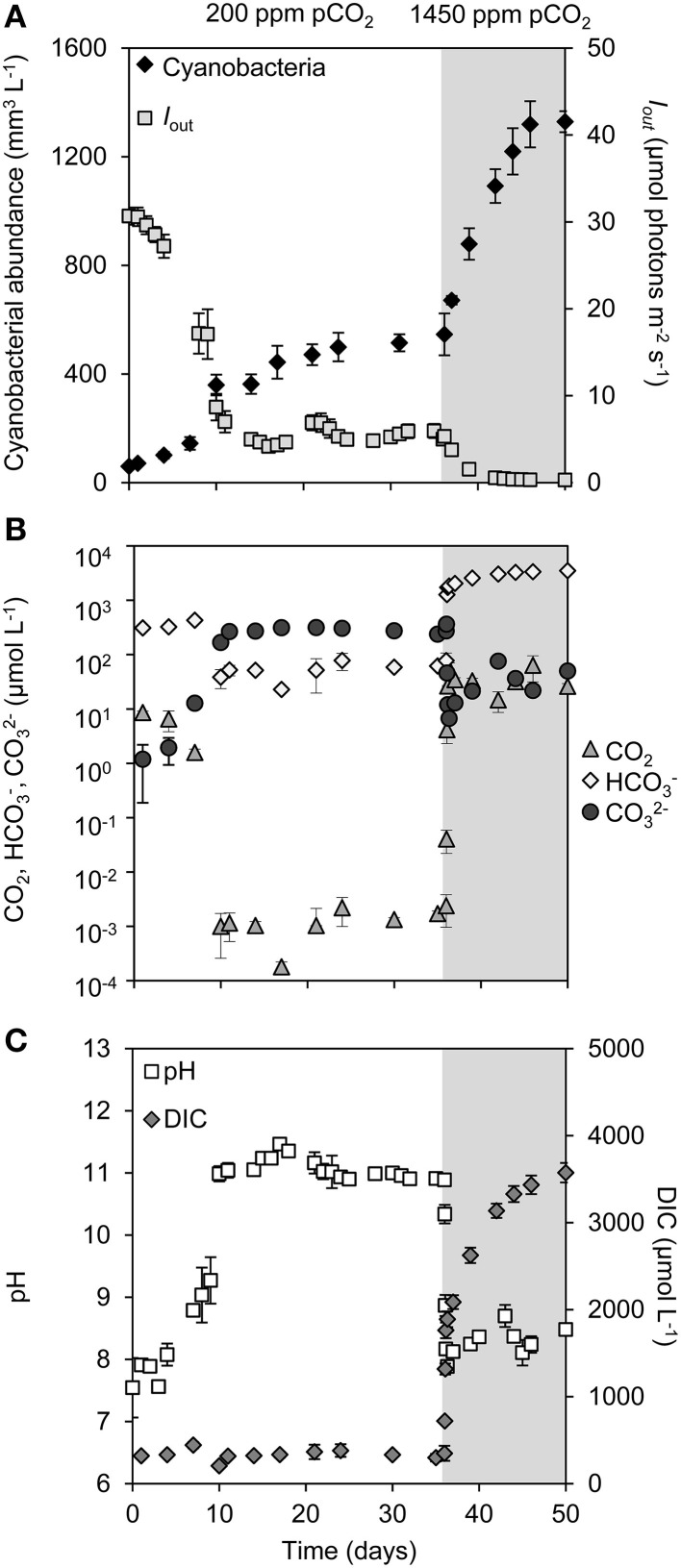
**Changes in cyanobacterial abundance, light, dissolved inorganic carbon (DIC) and pH during a shift from low pCO_2_ (200 ppm, *white area*) to high pCO_2_ (1450 ppm, *shaded area*)**. **(A)** Cyanobacterial abundance (expressed as biovolume) and light intensity penetrating through the chemostat (*I_out_*). **(B)** Dissolved CO_2_ (dCO_2_), bicarbonate (HCO^−^_3_) and carbonate (CO^2−^_3_) concentrations (logarithmic scale). **(C)** Dissolved inorganic carbon (DIC) and pH. Error bars indicate standard deviations (*n* = 4).

During the first phase of the experiment, we used a low pCO_2_ concentration in the gas flow of 200 ppm to create C_i_-limited conditions. In all four replicate chemostats, the cyanobacterial abundance gradually increased over time and approached a steady state after ~17 days (Figure 1A). Cell growth in the chemostats attenuated the light passing through, so that the light penetration *I_out_* at this first steady state was reduced to ~6 μmol photons m^−2^ s^−1^ (Figure 1A). The dCO_2_ concentration was depleted to the nano-molar range (Figure 1B). This was accompanied by a rise in pH to ~11, a strong decrease in bicarbonate concentration to ~60 μmol L^−1^ and a strong increase in carbonate concentration to ~300 μmol L^−1^ (Figures 1B,C). The total DIC concentration was not much affected during this first phase of the experiment (Figure 1C).

After 36 days, we raised the pCO_2_ level in the gas flow to 1450 ppm pCO_2_. The transition from 200 to 1450 ppm led to major changes in pH and inorganic carbon chemistry (Figures 1B,C; Supplementary Figure 1). Within 24 h, the dCO_2_ concentration increased over more than four orders of magnitude to ~30 μmol L^−1^, while the pH dropped back from 11 to 8 and the bicarbonate concentration increased to ~2000 μmol L^−1^ (Supplementary Figure 1). The enhanced CO_2_ availability alleviated the cyanobacteria from C_i_ limitation, thus enabling a strong increase in cyanobacterial abundance (Figure 1A). The resultant high biomass effectively absorbed all incident light, reducing light penetration through the chemostats to *I_out_* < 0.1 μmol photons m^−2^ s^−1^ (Figure 1A). Nitrate uptake by the growing cyanobacterial population increased alkalinity (Supplementary Figure 2), which initiated a progressive further increase of the DIC concentration (Figure 1C). After 46 days a new steady state was attained, with a ~2.7-fold higher cyanobacterial abundance than the previous steady state, a DIC concentration of ~3500 μmol L^−1^ consisting largely of bicarbonate and CO_2_, but low light conditions (Figures 1A–C).

### Cell volume, cell weight, and cellular composition

**Figure 2:**
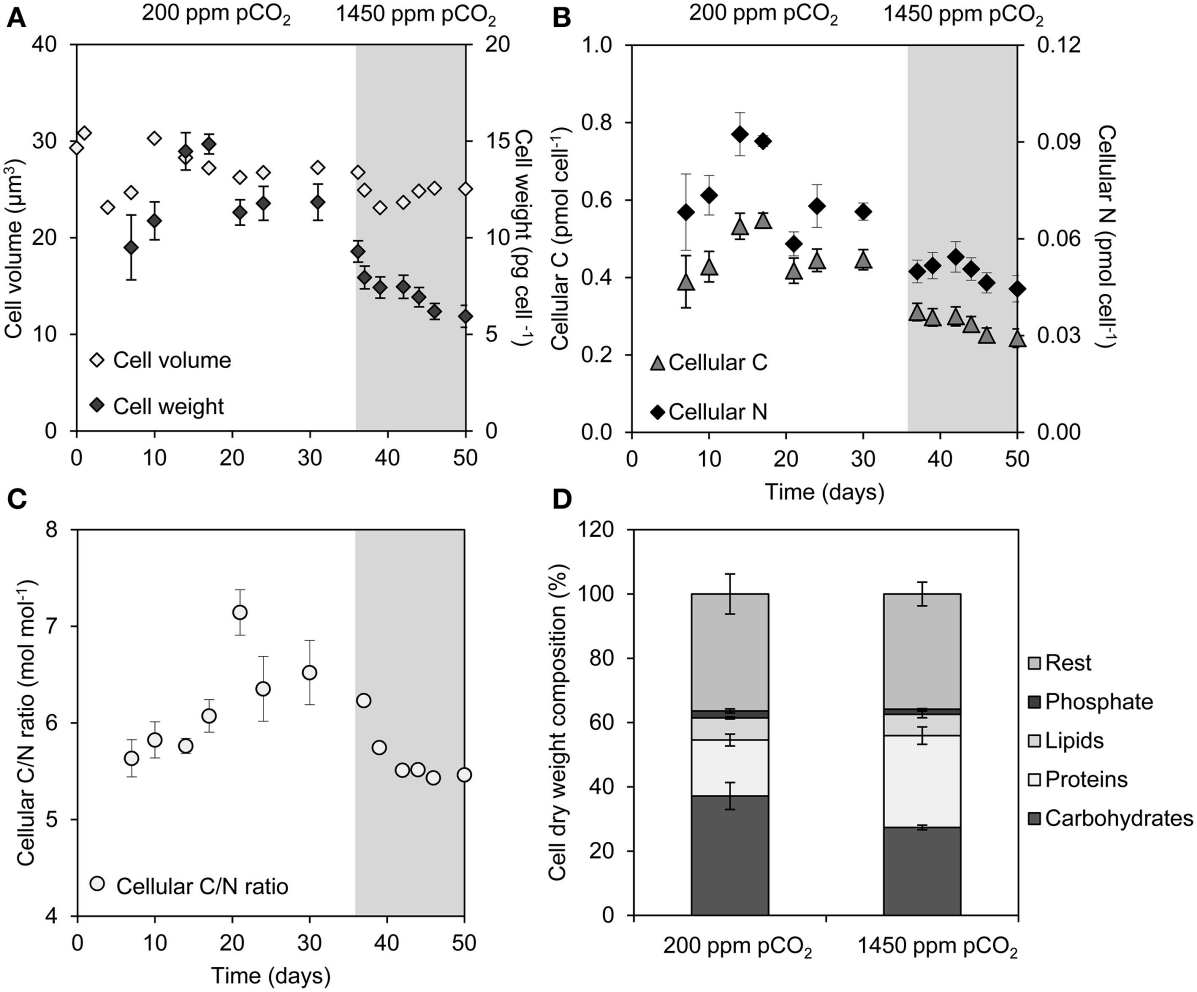
**Changes in cell properties during a shift from low pCO_2_ (200 ppm, *white area*) to high pCO_2_ (1450 ppm, *shaded area*)**. **(A)** Average cell volume and cell weight. **(B)** Cellular elemental C and N content. **(C)** Molar C/N ratio of the cells. **(D)** Dry weight composition of cells from the steady states at 200 and 1450 ppm pCO_2_. Error bars indicate standard deviations (*n* = 4).

Although the cell volume of *Microcystis* was hardly affected by the elevated pCO_2_, the average dry weight of the cells decreased by 50%, from 12 to 6 pg celL^−1^ (Figure 2A). This was accompanied by a decrease of the C and N content of the cells at elevated pCO_2_ (Figure 2B). Remarkably, the molar C/N ratio of the cells also slightly decreased at elevated pCO_2_ (Figure 2C), despite the strong increase in C_i_ availability.

Major macromolecules (carbohydrates, proteins, and lipids) and phosphate stored in the cells were quantified just before and 2 weeks after raising the pCO_2_ level (Figure 2D). The carbohydrate content (expressed as % dry weight) decreased significantly (Student *t*-test: *t*_6_ = 4.6, *p* < 0.01), whereas the protein content increased significantly at elevated pCO_2_ (Student *t*-test: *t*_6_ = -6.8, *p* < 0.001) (Figure 2D), which is consistent with the decrease of the cellular C/N ratio (Figure 2C). Lipids and phosphate contributed less to the total average cell weight than carbohydrates. Genes involved in carbohydrate storage and polyhydroxyalkanoate (PHA) storage showed no large changes in expression during the experiment, although minor upregulation of the *pha* genes was noticeable 8 h after the switch to high pCO_2_ (Table 1).

**Table 1:** **Genes responding to rising pCO_2_ and involved in pigment synthesis, photosystems, C metabolism, C storage, N assimilation, and stress response**.

**Gene or IPF number**	**Gene function category**	**Annotation**	**Log2 values at different time points (h)**						
			**0.75**	**2**	**4**	**8**	**24**	**72**	**336**
***PIGMENT GENES (3 OF THE 33)***									
*cpcI*	8	Phycocyanin synthesis	ns	ns	ns	ns	ns	0.93	0.92
*crtO*	2	Beta-carotene synthesis	ns	ns	ns	1.05	ns	ns	ns
1003	16	Beta-carotene synthesis	ns	0.94	0.99	ns	ns	ns	ns
***PHOTOSYSTEM GENES (0 OF THE 31)***									
***C METABOLISM GENES (2 OF THE 38)***									
*zwf*	6	Glycolysis and oxidative pentose	ns	ns	ns	0.90	ns	ns	ns
*icd*	6	Phosphate pathway citric acid cycle	ns	ns	ns	0.95	ns	ns	ns
***C STORAGE GENES (4 OF THE 15)***									
*phaC*	5	Polyhydroxyalkanoate storage	ns	ns	ns	1.13	ns	ns	ns
*phaA*	5	Polyhydroxyalkanoate storage	ns	ns	ns	1.18	ns	ns	ns
*phaB*	5	Polyhydroxyalkanoate storage	ns	ns	ns	1.15	ns	ns	ns
*phaE*	5	Polyhydroxyalkanoate storage	ns	ns	0.91	1.20	ns	ns	ns
***N ASSIMILATION GENES (9 OF THE 29)***									
*glnN*	1	GS/GOGAT	ns	1.24	1.51	0.94	ns	1.16	1.45
*gltB*	1	GS/GOGAT	ns	ns	ns	0.91	ns	ns	ns
*nrtA*	14	Nitrate transport	ns	1.08	1.02	ns	ns	ns	ns
*nrtB*	14	Nitrate transport	ns	0.99	ns	ns	ns	ns	ns
*ntcB*	1	Nitrate transport	ns	1.05	0.90	ns	ns	ns	ns
*nirA*	1	Nitrite reductase	ns	0.92	ns	ns	ns	ns	ns
1263	14	Ammonium transport	ns	1.15	1.44	0.97	ns	ns	ns
*urtA*	14	Urea transport	ns	ns	0.94	ns	ns	ns	ns
5363	14	Urea transport	ns	0.91	1.01	ns	ns	ns	ns
***STRESS-RELATED GENES (8 OF THE 22)***									
*flv2*	15	Flavoprotein	ns	ns	−1.81	ns	ns	ns	ns
*flv4*	15	Flavoprotein	ns	ns	−2.31	ns	ns	ns	ns
*isiA*	8	Chlorophyll-binding	ns	ns	−1.47	−1.33	ns	ns	−1.76
*isiB*	8	Flavodoxin	ns	ns	−1.03	ns	ns	ns	ns
*sigB*	12	Sigma factor	ns	ns	ns	ns	ns	−1.16	ns
*sigE*	12	Sigma factor	ns	ns	ns	0.94	ns	ns	ns
*sigH*	12	Sigma factor	ns	ns	−1.30	−1.36	−1.59	−2.49	−2.62

*Log2 values quantify gene expression at the given time point (after increasing the pCO_*2*_) with respect to gene expression at t = 0 (just before increasing the pCO_*2*_). Only the responsive genes are shown; the complete data set including all non-responsive genes is provided in Supplementary Table 4. IPF numbers are the locus tags of Microcystis PCC 7806 in the EMBL database (AM778843–AM778958). ns, not significant; GS/GOGAT, glutamine synthetase/glutamine-2-oxoglutarate amidotransferase; Gene function categories (CyanoBase), (1) amino acid biosynthesis; (2) biosynthesis of cofactors, prosthetic groups, and carriers; (5) central intermediary metabolism; (6) energy metabolism; (8) photosynthesis, and respiration; (12) transcription; (14) transport and binding proteins; (15) other categories; (16) hypothetical*.

### Pigments and photosystems

**Figure 3:**
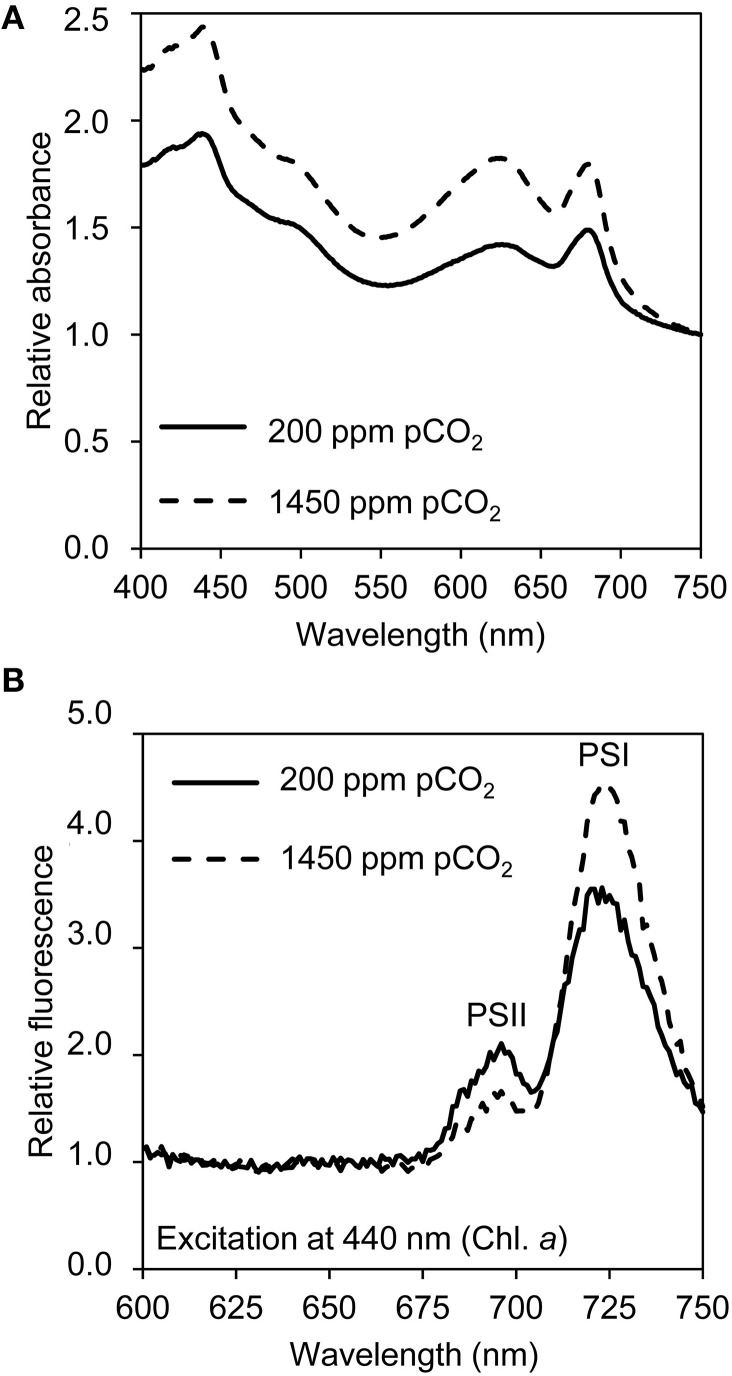
**Light absorption and emission spectra of cells grown at low pCO_2_ (200 ppm) and high pCO_2_ (1450 ppm)**. **(A)** Light absorption spectra normalized at 750 nm, with peaks of chlorophyll *a* (436 and 678 nm), β-carotene (shoulder at 490 nm) and phycocyanin (626 nm). **(B)** 77 K fluorescence emission spectra normalized based on the mean emission at 600–660 nm, with peaks of PSI (720 nm) and PSII (695 nm). The spectra are the average of four biological replicates.

Light absorption spectra normalized at 750 nm indicated that the cellular contents of the pigments chlorophyll *a*, phycocyanin, and β-carotene increased at elevated pCO_2_ (Figure 3A). The phycocyanin peak (at 626 nm) increased slightly more than the chlorophyll *a* peak (at 678 nm). The ratio of PSI to PSII changed, with less PSII and more PSI at elevated pCO_2_ (Figure 3B). Yet, only a few genes involved in pigment synthesis and none of the photosystem genes changed expression significantly after exposure to high pCO_2_ (Table 1).

### Secondary metabolites including toxins

**Figure 4:**
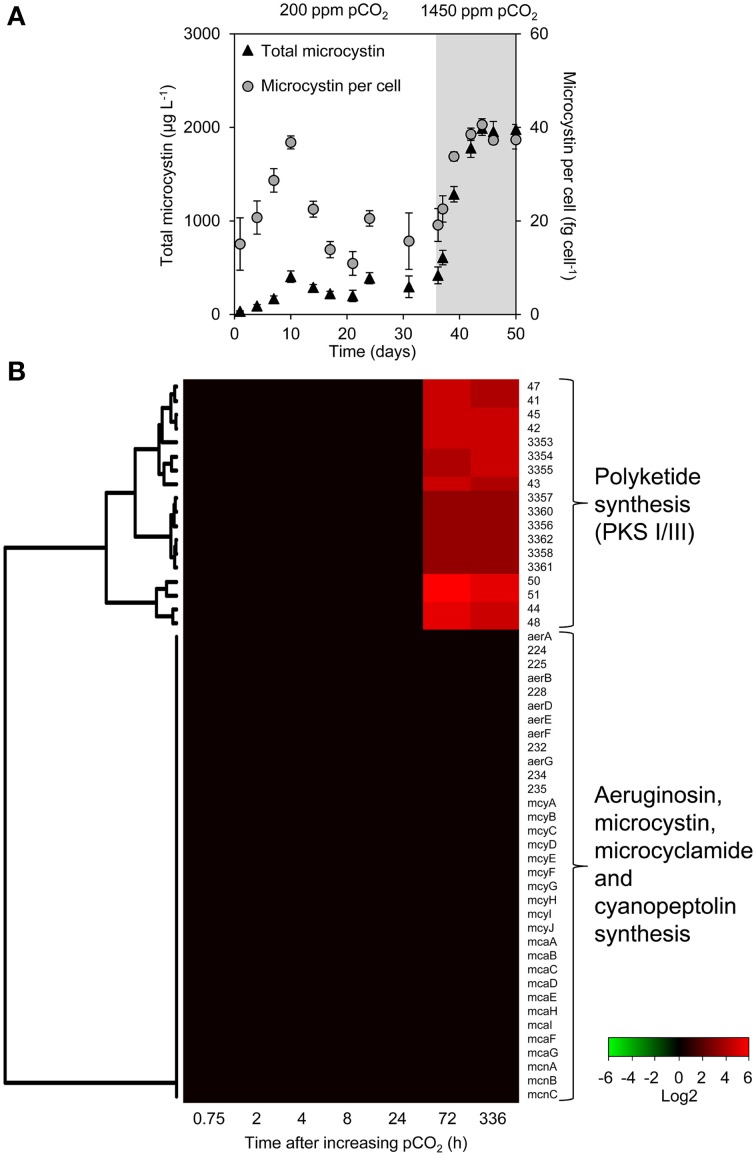
**Changes in microcystin concentration and gene expression of secondary metabolite genes**. **(A)** Total microcystin concentration and microcystin content per cell, during the shift from low pCO_2_ (200 ppm, *white area*) to high pCO_2_ (1450 ppm, *shaded area*). Error bars indicate standard deviations (*n* = 4). **(B)** Changes in expression of the secondary metabolite genes after the increase of pCO_2_ to 1450 ppm. Expression changes are quantified as log2 values. Red indicates significant upregulation and green significant downregulation; non-significant changes are in black. Hierarchical clustering was used to order the genes. The underlying data are presented in Supplementary Table 4.

The microcystin concentration stabilized at ~300 μg L^−1^ at low pCO_2_ and increased 6-fold to ~2000 μg L^−1^ at high pCO_2_ (Figure 4A). The microcystin content per cell was ~15 fg celL^−1^ at the steady state at low pCO_2_, and increased to ~38 fg celL^−1^ at high pCO_2_. Other secondary metabolites produced by *Microcystis* PCC 7806 include aeruginosin, microcyclamide, cyanopeptolin, and polyketides. However, expression of the microcystin, aeruginosin, microcyclamide, and cyanopeptolin operons did not change significantly (Figure 4B). In contrast, the expression of two polyketide synthase operons (PKS I/III) increased strongly 72 and 336 h after increasing the pCO_2_.

### Carbon, nitrogen and stress-related genes

**Figure 5:**
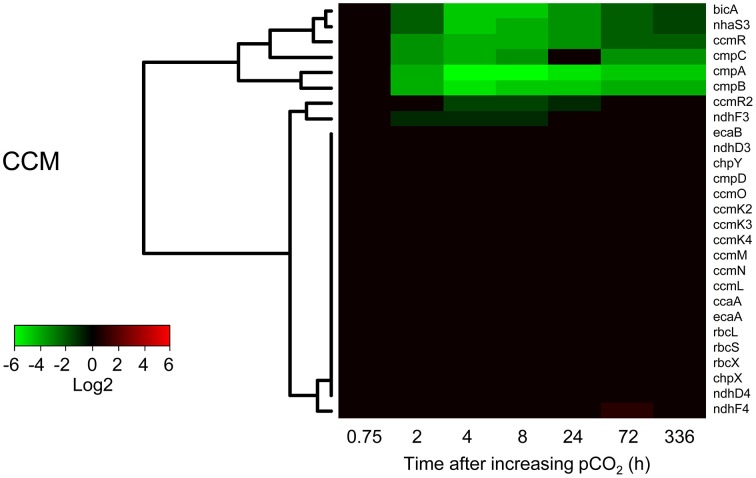
**Changes in expression of the CO_2_-concentrating mechanism (CCM) genes after the increase of pCO_2_ to 1450 ppm**. Expression changes are quantified as log2 values. Red indicates significant upregulation and green significant downregulation; non-significant changes are in black. Hierarchical clustering was used to order the genes. The underlying data are presented in Supplementary Table 4.

Several but not all genes involved in the CCM of *Microcystis* responded to elevated CO_2_ (Figure 5). Expression of the *cmpABCD* operon encoding the BCT1 bicarbonate uptake system and the *bicA*-*nhaS3* operon encoding the BicA bicarbonate uptake system was strongly reduced after 2 h at high pCO_2_ and remained low during the next 2 weeks. Expression levels of the transcriptional regulators *ccmR* (upstream of the high-affinity CO_2_ uptake operon) and *ccmR2* (upstream of the *bicA*-*nhaS3* operon) were also downregulated at elevated pCO_2_. In contrast, expression of the CO_2_ uptake system genes remained unchanged, except for a slight downregulation of the *ndhF3* gene involved in high-affinity CO_2_ uptake. Expression of genes for carboxysome formation (*ccmK2-4, ccmL, cmmM, ccmN*, and *ccmO*), RuBisCO (*rbcL, rbcS*, and *rbcX*) and carbonic anhydrases (*ccaA, ecaA*, and *ecaB*) remained constant as well (Figure 5). Results of the microarray experiments were validated with RT-qPCR for selected CCM genes, which gave similar results (Supplementary Figure 3).

Only a few genes involved in C metabolism and N assimilation showed significant changes in expression at elevated pCO_2_ (Table 1). Expression of *glnN*, encoding for a glutamine synthetase, was upregulated at elevated pCO_2_. The genes *nrtA* and *nrtB* involved in nitrate uptake, *nirA* involved in nitrite reduction, and *ntcB* involved in the control of N assimilation were slightly upregulated shortly after increasing the pCO_2_.

Several stress-related genes were downregulated at elevated pCO_2_, including the flavodiiron protein genes *flv2* and *flv4*, the iron-stress chlorophyll-binding protein gene *isiA* and the sigma factor *sigH* (Table 1).

### Physiological assay of C_i_ uptake

**Figure 6:**
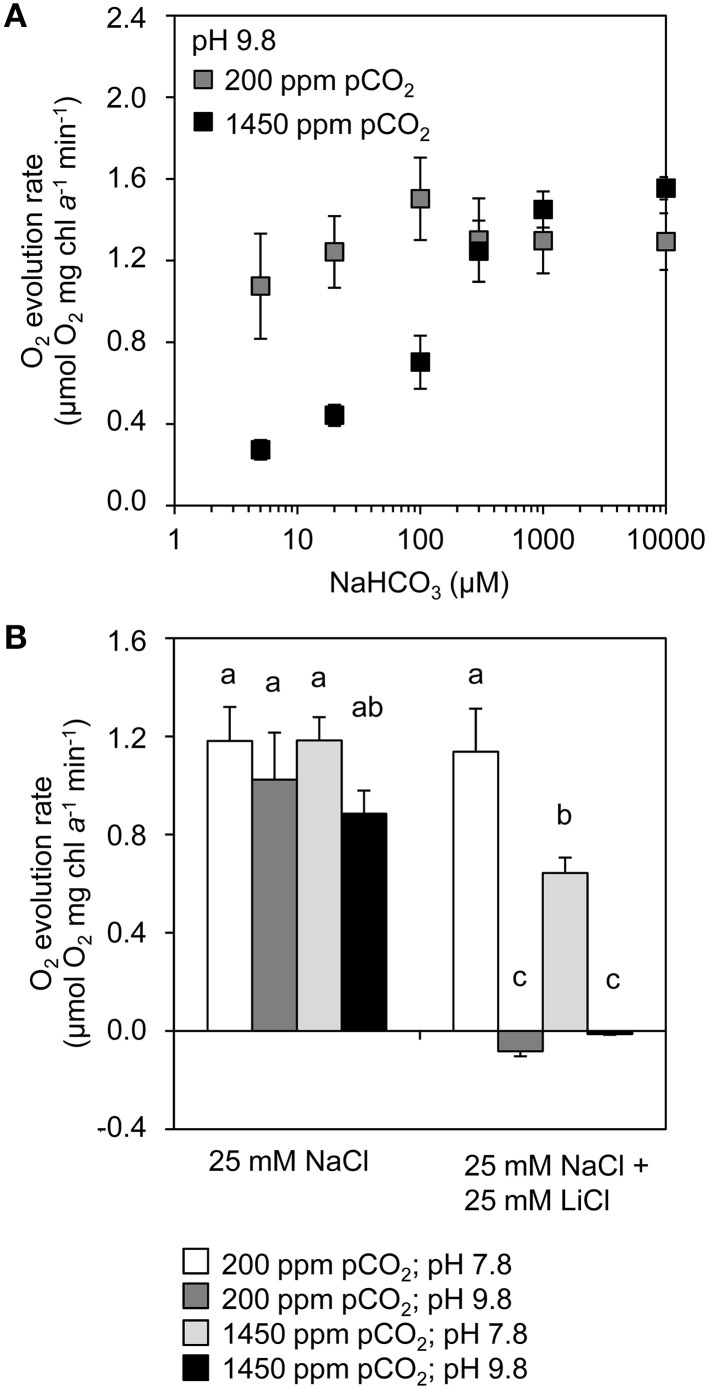
**Inorganic carbon uptake kinetics of cells grown at low pCO_2_ (200 ppm) and high pCO_2_ (1450 ppm)**. **(A)** Bicarbonate response curves (O_2_ evolution rates) of cells grown at low vs. high pCO_2_ after addition of different concentrations of NaHCO_3_ at pH 9.8. **(B)** O_2_ evolution rates in the presence and absence of LiCl, at pH 7.8 and pH 9.8. The cells were provided with 200 μmol L^−1^ KHCO_3_. Lithium ions block sodium-dependent bicarbonate uptake, while differences in pH produce different concentrations of dCO_2_ and bicarbonate. Error bars indicate standard deviations (*n* = 4). Bars with different letters were significantly different, as tested by a one-way analysis of variance with *post-hoc* comparison of the means (α = 0.05).

To assess the activity of the bicarbonate uptake systems BicA and BCT1, O_2_ evolution was studied as a function of bicarbonate availability (Figure 6A). We added 25 mmol L^−1^ NaCl to enable sodium-dependent bicarbonate uptake by BicA. The experiments were performed at pH 9.8, at which DIC contains only ~0.03% dCO_2_. Without DIC in the medium the samples did not evolve O_2_, but their O_2_ evolution resumed after addition of NaHCO_3_. Cells from the chemostats at 200 ppm pCO_2_ had a higher affinity at low NaHCO_3_ concentrations (5–100 μmol L^−1^) than cells from the chemostats at 1450 ppm pCO_2_. At higher NaHCO_3_ concentrations (300–10,000 μmol L^−1^), cells from both pCO_2_ conditions reached a plateau in O_2_ evolution (Figure 6A). These results suggest that the high-affinity bicarbonate uptake system BCT1 was active in the chemostats at low pCO_2_ (200 ppm) but not at elevated pCO_2_ (1450 ppm), while the low-affinity bicarbonate uptake system BicA was active at both pCO_2_ levels.

To further explore the involvement of the C_i_ uptake systems, we added lithium ions to inhibit the sodium-dependent bicarbonate uptake of BicA (Figure 6B). These experiments were performed at pH 7.8 and pH 9.8, at which DIC contains 3.7% and 0.03% dCO_2_, respectively. For cells taken from both low and high pCO_2_ chemostats, we found high activity at pH 7.8 and at pH 9.8. However, addition of 25 mmol L^−1^ LiCl resulted in high activity at pH 7.8 but not at pH 9.8. At pH 7.8, the conversion of bicarbonate to CO_2_ and subsequent uptake by the CO_2_ uptake systems likely compensated for the inactivation of BicA by lithium ions. In contrast, at pH 9.8, when dCO_2_ is negligible and C_i_ uptake relies on bicarbonate, the inactivation of BicA by lithium ions was not compensated and drastically reduced C_i_ uptake. These results suggest that, in the chemostats, both BicA and the CO_2_ uptake systems were active at both pCO_2_ conditions.

## Discussion

### Alleviation from C_i_ limitation

During dense blooms in eutrophic and hypertrophic lakes, cyanobacterial growth can become limited by a low C_i_ availability (Ibelings and Maberly, 1998; Balmer and Downing, 2011; Verspagen et al., 2014b). Our experiments show that at low atmospheric pCO_2_ levels, the photosynthetic activity of the cyanobacteria depleted the dCO_2_ concentration to the nanomolar range, while the bicarbonate and carbonate concentrations were orders of magnitude higher, which forced cells to primarily use bicarbonate as carbon source. An increase in pCO_2_ shifted the growth conditions from very low dCO_2_ concentrations and modest light availability to higher dCO_2_ concentrations but lower light levels (Figure 1). The *Microcystis* population in our experiments benefited strongly from the increased dCO_2_ availability, and accelerated cell division during the subsequent transient phase. This demonstrates that growth was indeed C_i_ limited at low pCO_2_ levels. The increased cyanobacterial abundance reduced light availability, until the cyanobacterial population settled at a new steady state. Hence, our experiments support the prediction (Verspagen et al., 2014b) that rising CO_2_ levels will alleviate cyanobacteria from C_i_ limitation, and are likely to increase the cyanobacterial abundance in eutrophic and hypertrophic lakes.

Surprisingly, the increase in pCO_2_ induced changes in the expression of only 234 genes of *Microcystis* PCC 7806 (Supplementary Figure 4). This is only ~5% of the total number of protein-encoding genes investigated in this study, and many of these showed differential expression at only a few time points. For instance, only 0.6% of the genes were differentially expressed at the time point of 24 h after the rise in CO_2_. In contrast, in other studies, 20–25% of the genes of *Synechocystis* PCC 6803 (Eisenhut et al., 2007) and 50–55% of the genes of *Synechococcus elongatus* PCC 7942 (Schwarz et al., 2011) were differentially expressed after 24 h of C_i_ limitation.

The much stronger transcriptome response in these two latter studies than in our results might be explained by differences in experimental design. (Eisenhut et al., 2007; Schwarz et al., 2011) exposed the cells to C_i_ starvation, which ceased cell growth and induced a large stress response. In contrast, the growth rate in our chemostat experiments was kept relatively high, which resulted in a milder response to elevated CO_2_. An alternative explanation might be that the cyanobacterium *Microcystis* shows a much more specific transcriptome response to changing C_i_ conditions than *Synechocystis* and *Synechococcus*. *Microcystis* is a buoyant cyanobacterium that can develop dense blooms in eutrophic lakes, where it is typically exposed to a wide range of different CO_2_ conditions (Verspagen et al., 2014b). A highly specific transcriptome response that mainly targets the C_i_ uptake systems could preserve energy and offer a robust strategy for a species that often experiences strongly fluctuating C_i_ conditions.

### Changes in expression of CCM genes

Our results show that the bicarbonate uptake genes encoding for BCT1 and BicA were downregulated at elevated pCO_2_ (Figure 5). Additionally, expression of the transcriptional regulator *ccmR2*, located upstream of the *bicA*-*nhaS3* operon, was lowered.

BCT1 has a relatively high affinity for bicarbonate (K_0.5 ~10–15_ μmol L^−1^) and a low flux rate (Omata et al., 2002). In line with expectation, expression of the genes *cmpABCD* encoding BCT1 was highest at the low pCO_2_ condition. This is further supported by the bicarbonate response curves of the O_2_ optode experiments, which showed high-affinity bicarbonate uptake at low pCO_2_ but not at high pCO_2_ (Figure 6A). The high activity of BCT1 in low pCO_2_ conditions is consistent with previous studies with other cyanobacteria, which showed enhanced expression of the *cmpABCD* genes upon induction of C_i_ limitation (Woodger et al., 2003; Eisenhut et al., 2007; Schwarz et al., 2011).

Conversely, BicA has a relatively low affinity for bicarbonate (K_0.5_ ~70–350 μmol L^−1^) while it has a high flux rate (Price et al., 2004). Contrary to expectation, the *bicA* expression was highest at low pCO_2_, where the bicarbonate concentration was only ~60 μmol L^−1^ (Figure 1B). This was also observed in a study of *Synechococcus* PCC 7002, where the expression of *bicA* was enhanced by C_i_ limitation (Woodger et al., 2007). However, it differs from studies of *Synechocystis* PCC 6803, which found that the expression of *bicA* was not much affected by the pCO_2_ level (Wang et al., 2004; Eisenhut et al., 2007). Our results show that, even though the *bicA* gene of *Microcystis* PCC 7806 was less expressed at high pCO_2_, the BicA enzyme was still active at high pCO_2_. This is apparent from the O_2_ optode experiments in which we blocked BicA activity with lithium ions (Figure 6). Hence, BicA appears active at a relatively wide range of bicarbonate concentrations.

Compared to several other *Microcystis* strains, *Microcystis* PCC 7806 lacks the bicarbonate uptake system SbtA (Sandrini et al., 2014), which has an even higher affinity for bicarbonate (K_0.5_ < 2 μmol L^−1^) than BCT1 (Price et al., 2004). Therefore, *Microcystis* PCC 7806 seems less adapted to very low bicarbonate conditions than SbtA-containing *Microcystis* strains, and was likely trying to take up as much bicarbonate as possible by expressing both BCT1 and BicA at the low pCO_2_ condition, even though the BicA uptake system did not operate very efficiently at that stage.

In contrast to the strong response of the bicarbonate uptake genes, gene expression of the CO_2_ uptake systems was not affected by elevated pCO_2_ (Figure 5). For instance, the *chpX* and *chpY* genes, encoding for the CO_2_ hydration subunit of the low-affinity and high-affinity CO_2_ uptake systems, respectively, were constitutively expressed in *Microcystis* PCC 7806. The constitutive expression of *chpX* is consistent with studies of other cyanobacteria (Woodger et al., 2003; Wang et al., 2004; Schwarz et al., 2011). However, the constitutive expression of *chpY* contrasts with studies of other cyanobacteria, which found that *chpY* was highly inducible when cells were exposed to C_i_ limitation (Price et al., 2004; Wang et al., 2004; Schwarz et al., 2011). Constitutive expression of the CO_2_ uptake systems indicates that at low dCO_2_ concentrations the *Microcystis* cells were still capable of CO_2_ uptake. This was confirmed by the O_2_ optode experiments, which showed that CO_2_ uptake was active at both pCO_2_ levels when bicarbonate uptake was blocked by lithium ions (Figure 6B).

Expression of the transcriptional regulator *ccmR*, located upstream of the high-affinity CO_2_ uptake operon, was strongly downregulated at high pCO_2_ (Figure 5). This is noteworthy, because expression of the high-affinity CO_2_ uptake genes did not respond to elevated pCO_2_. Possibly, CcmR regulates the expression of the *cmpABCD* operon of *Microcystis*, which was strongly downregulated at high pCO_2_. This would be consistent with the observation that CmpR, a known transcriptional regulator of the *cmpABCD* operon in other cyanobacteria, is lacking in *Microcystis* (Sandrini et al., 2014). Indeed, Woodger et al. (2007) previously showed that CcmR in *Synechococcus* PCC 7002 does not only control the high-affinity CO_2_ uptake operon but also the expression of other CCM genes.

Interestingly, gene expression of several key components of the CCM, including carboxysome, RuBisCO and carbonic anhydrase genes, was not affected by elevated pCO_2_ (Figure 5), suggesting that cells did not alter carboxysome component numbers. This is counterintuitive, since C_i_ availability strongly increased after the upshift in pCO_2_. Yet, it seems that *Microcystis* simply adjusted its C_i_ uptake arsenal to deal with the change in C_i_ availability. In contrast, studies that induced C_i_ limitation in *Synechocystis* PCC 6803 and *Synechococcus elongatus* PCC 7942 observed downregulation of carboxysomal genes after 24 h (Eisenhut et al., 2007; Schwarz et al., 2011). Possibly, the cyanobacteria in these latter studies did not need to produce new carboxysomes, because their growth rate was arrested in the experiments of Eisenhut et al. (2007) and Schwarz et al. (2011). This differs from our chemostat experiments, in which the growth rate was kept relatively high by the dilution rate, and hence carboxysome production should be maintained. Therefore, differences in experimental design might explain these results. Alternatively, the contrasting results might reflect species-specific differences, where *Microcystis* displays a milder response to fluctuating C_i_ conditions than these other cyanobacteria (see preceding discussion in section Alleviation from C_i_ limitation).

### Reduced expression of stress-related genes

Our results show reduced expression of several stress genes (*flv2, flv4, isiA, sigH*) at elevated CO_2_ (Table 1). Flavodiiron proteins have previously been linked to acclimation to low C_i_ conditions (Zhang et al., 2009; Allahverdiyeva et al., 2011; Bersanini et al., 2014). Flv2 and Flv4 are unique for cyanobacteria. They are involved in photoprotection of the PSII complex (Zhang et al., 2009), and can function as electron sink (Bersanini et al., 2014). Reduced expression of the flavodiiron protein genes *flv2* and *flv4* at elevated pCO_2_ is in agreement with previous studies with *Synechocystis* PCC 6803 (Wang et al., 2004; Eisenhut et al., 2007).

The iron-stress chlorophyll-binding protein IsiA can function as a storage unit for spare chlorophyll molecules, as a PSI antenna enhancing the light harvesting ability, and in addition can protect PSII from photooxidative stress (Yeremenko et al., 2004; Havaux et al., 2005). Reduced expression of the *isiA* gene at elevated pCO_2_ likely signifies lower levels of oxidative stress, which shifted the IsiA-bound spare chlorophylls to new chlorophyll protein complexes.

Sigma factors are involved in transcription initiation. *sigH* expression typically increases with general stress (Huckauf et al., 2000; Hakkila et al., 2013). The expression of *sigH* was lowered in our study after the increase in pCO_2_ (Table 1), indicating that the cells perceived less stress at this stage.

### Increased toxins per cell at elevated CO_2_?

Our results show that cells of *Microcystis* PCC 7806 contained a 2.5 times higher microcystin content at elevated pCO_2_. Similar results were found in an earlier study with *Microcystis* HUB 5-2-4, where elevated CO_2_ also produced a transition from C_i_ to light limitation and an ~2–2.5-fold increase in microcystin content (Van de Waal et al., 2009). This suggests that rising CO_2_ levels will increase the toxicity of *Microcystis* cells.

In contrast to the increase of the cellular microcystin content, expression of the *mcy* genes remained constant during the shift from low pCO_2_ to high pCO_2_ (Figure 4B). Gene expression may not be equivalent to abundance of the final gene product, and a further complication is that microcystins are synthesized by non-ribosomal peptide synthetases (Dittmann et al., 1997). The control mechanisms for the synthetase activity are largely unknown, but it has been suggested that amino acid availability may play a decisive role (Tonk et al., 2008; Van de Waal et al., 2010a).

The microarray results showed a strong increase in two polyketide synthase operons (PKS I/III) at elevated pCO_2_ (Figure 4B). These operons were described previously (Frangeul et al., 2008; Makower et al., 2015), yet the structure of the actual produced compounds and the exact function remain unknown. Polyketides have diverse biological functions and many have pharmacological and toxicological properties. Further research is needed to know more about the polyketides of cyanobacteria.

### Other physiological responses to elevated CO_2_

Our results also point at several other physiological changes in response to rising pCO_2_ levels. For example, the dry weight of cells decreased ~2-fold at elevated pCO_2_, but the cells were hardly smaller (Figure 2A). This was accompanied by a reduced cellular C storage, lower cellular C/N ratio and reduced carbohydrate content at elevated pCO_2_ (Figures 2B–D). A lower cellular C content at elevated CO_2_ contradicts the common expectation that rising CO_2_ levels will increase the carbon content of phytoplankton cells (e.g., Finkel et al., 2010; Van de Waal et al., 2010b; Verschoor et al., 2013). However, recent experiments showed that also other *Microcystis* strains do not increase their C/N ratio at elevated CO_2_ when provided with an ample nutrient supply (Verspagen et al., 2014a). A possible explanation for these observations might be that cells do not require large amounts of C reserves in the presence of plentiful C_i_ in the environment. Furthermore, changes in carbohydrate content are known to play a role in the vertical migration of *Microcystis* (Thomas and Walsby, 1985; Visser et al., 1997; Wallace et al., 2000). A strong decrease in cellular dry matter and carbohydrate content will increase the buoyancy of cells, which may be particularly advantageous when cells experience low light levels. The shift from C_i_ to light limitation induced by rising CO_2_ concentrations may thus stimulate the formation of surface blooms in *Microcystis*-dominated lakes.

Elevated pCO_2_ levels also led to an increase in the photosynthetic pigments and in the PSI/PSII ratio of the cells (Figures 3A,B). Most likely, this response is due to the reduced light availability caused by the denser cultures at elevated pCO_2_. This process, known as photoacclimation, is well-known to occur in cyanobacteria (Deblois et al., 2013). Yet, expression of the *psa* and *psb* genes for photosystem components remained unaltered.

We did not observe many changes in the genes involved in C and N metabolism (Table 1). One exception is the upregulation of *glnN*, that encodes for a glutamine synthase. The expression of *glnN* has been linked with nitrogen stress (Reyes et al., 1997). In this way the N metabolism of the cells responds subtly to changes in C_i_ availability caused by high pCO_2_ levels.

## Concluding remarks

We investigated the genetic and physiological response of the harmful cyanobacterium *Microcystis* to rising CO_2_. The experiments were conducted in chemostats, which provide ideal conditions to study microorganisms in a controlled environment. Yet, chemostats provide a simplified experimental setting in comparison to the complexity of the natural world. In lakes, buoyancy regulation enables *Microcystis* populations to form dense blooms at the water surface (Reynolds and Walsby, 1975; Huisman et al., 2004), where they intercept the influx of atmospheric CO_2_ (Ibelings and Maberly, 1998). Hence, the formation of surface blooms is a successful strategy to avoid low CO_2_ availability deeper in the water column (Paerl and Ustach, 1982). However, despite their proximity to the surface, dissolved CO_2_ concentrations can be strongly depleted and pH may exceed 9 during surface blooms of buoyant cyanobacteria (López-Archilla et al., 2004; Verspagen et al., 2014b). The high concentration of cells in blooms causes a high local demand for inorganic carbon and consequently extreme carbon limitation (Ibelings and Maberly, 1998), resembling the conditions in our CO_2_-limited laboratory experiments.

In our experiments, elevated CO_2_ alleviated *Microcystis* cells from C_i_ limitation. In response, *Microcystis* induced changes in the expression of a relatively limited number of genes, including several genes involved in C_i_ uptake. The expression of several stress-related genes was reduced at elevated CO_2_, while only a few key genes at control sites of cellular C/N metabolism were regulated. At elevated CO_2_, *Microcystis* cells halted its high-affinity bicarbonate uptake systems, and relied on CO_2_ and low-affinity bicarbonate uptake systems. This supports earlier results that high-affinity bicarbonate uptake systems will become less essential with rising CO_2_ (Sandrini et al., 2014). This may ultimately lead to an evolutionary loss of high-affinity C_i_ uptake systems, as shown in evolution experiments with the green alga *Chlamydomonas reinhardtii*. This green alga lost its ability to induce high-affinity C_i_ uptake while maintaining its low-affinity C_i_ uptake after 1000 generations at elevated CO_2_ levels (Collins and Bell, 2004; Collins et al., 2006).

In addition to changes in C_i_ uptake, elevation of the CO_2_ concentrations in our experiments also led to a higher population abundance, increased buoyancy of the cells, and higher toxin content per *Microcystis* cell. This indicates that the current rise in atmospheric CO_2_ levels may strongly increase the problems associated with the harmful cyanobacterium *Microcystis* in eutrophic lakes.

### Conflict of interest statement

The authors declare that the research was conducted in the absence of any commercial or financial relationships that could be construed as a potential conflict of interest.
