# Supplementary material for: Changes in gene expression, cell physiology and toxicity of the harmful cyanobacterium Microcystis aeruginosa at elevated CO2
Source: Front Microbiol. 2015 May 5;6:401. doi: 10.3389/fmicb.2015.00401 (PMC4419860; doi:10.3389/fmicb.2015.00401)
Supplement: Supplementary file 1 [file DataSheet1.PDF]

10-04-2015

Supplementary Material

Changes in gene expression, cell physiology and toxicity of the harmful cyanobacterium *Microcystis aeruginosa* at elevated CO_2

Giovanni Sandrini[1], Serena Cunsolo[1], J. Merijn Schuurmans[1,2], Hans C. P. Matthijs[1], Jef Huisman[1]*

[1]Department of Aquatic Microbiology, Institute for Biodiversity and Ecosystem Dynamics, University of Amsterdam, Amsterdam, The Netherlands
[2]Department of Aquatic Ecology, Netherlands Institute of Ecology, Wageningen, The Netherlands

*** Correspondence:** Jef Huisman, Department of Aquatic Microbiology, Institute for Biodiversity and Ecosystem Dynamics, University of Amsterdam, P.O. Box 94248, 1090 GE Amsterdam, The Netherlands.
j.huisman@uva.nl

1. Supplementary Figures

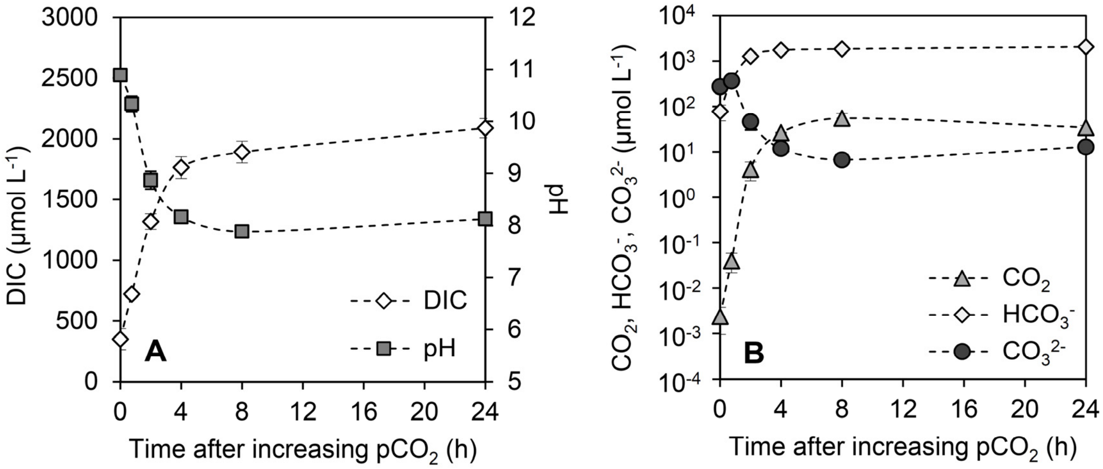

Supplementary Figure 1 Changes in inorganic carbon chemistry and pH during the first 24 h after increasing the pCO_2. **(A)** Dissolved inorganic carbon (DIC) and pH. **(B)** Dissolved CO_2, bicarbonate and carbonate concentrations. Error bars indicate standard deviations (*n* = 4).

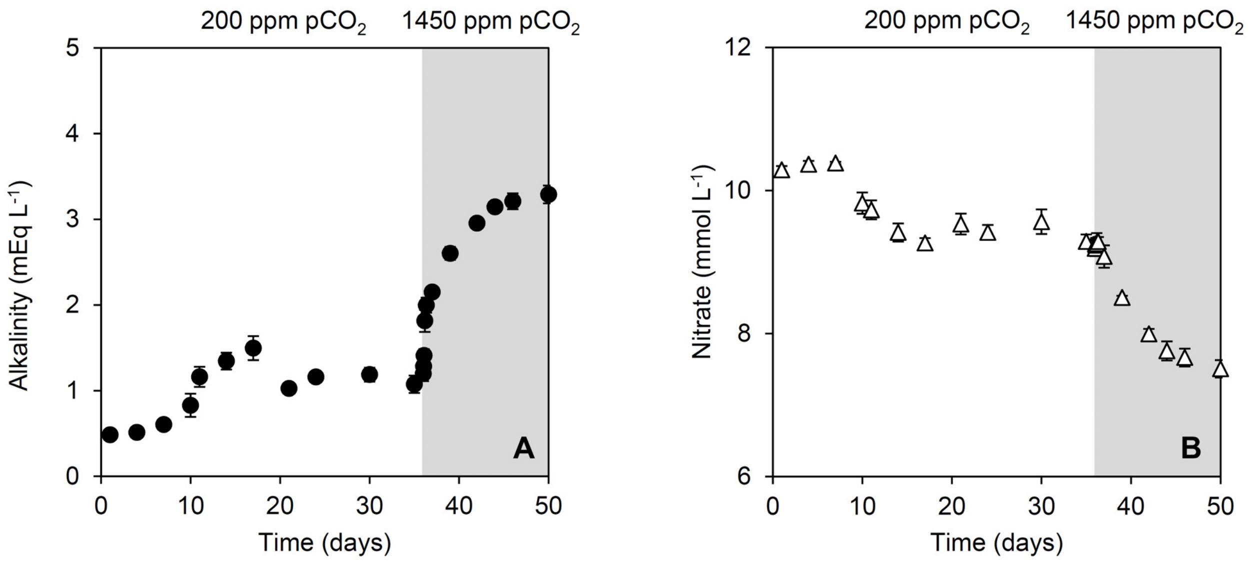

Supplementary Figure 2 Changes in alkalinity and nitrate concentration during the shift from low pCO_2 (200 ppm, *white area*) to high pCO_2 (1450 ppm, *shaded area*). **(A)** Alkalinity. **(B)** Nitrate concentration. Error bars indicate standard deviations (*n* = 4).

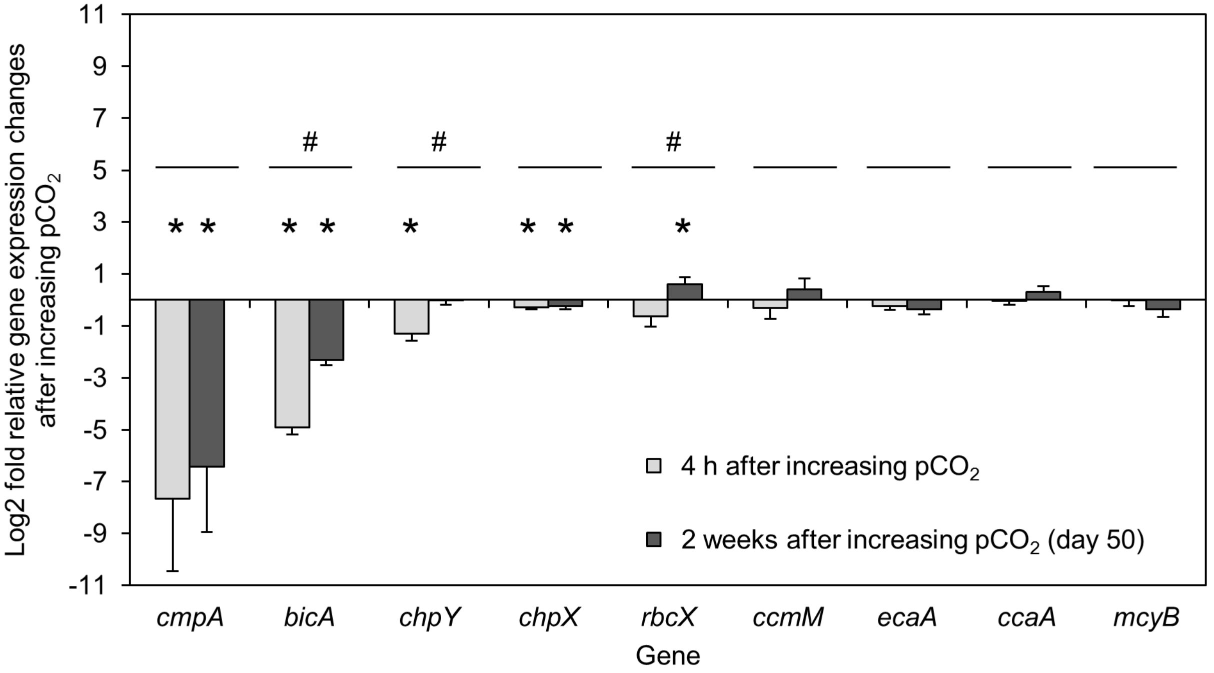

Supplementary Figure 3 Changes in gene expression of CCM genes and the microcystin gene *mcyB* assessed with RT-qPCR, at 4 h and 2 weeks after the shift from low pCO_2 (200 ppm) to high pCO_2 (1450 ppm). Error bars indicate standard deviations ($n = 4$). * significantly different ($p < 0.05$) from the zero time point (200 ppm). # significant difference ($p < 0.05$) between the time points of 4 h and 2 weeks after the increase to 1450 ppm. The p-values were corrected for multiple hypothesis testing.

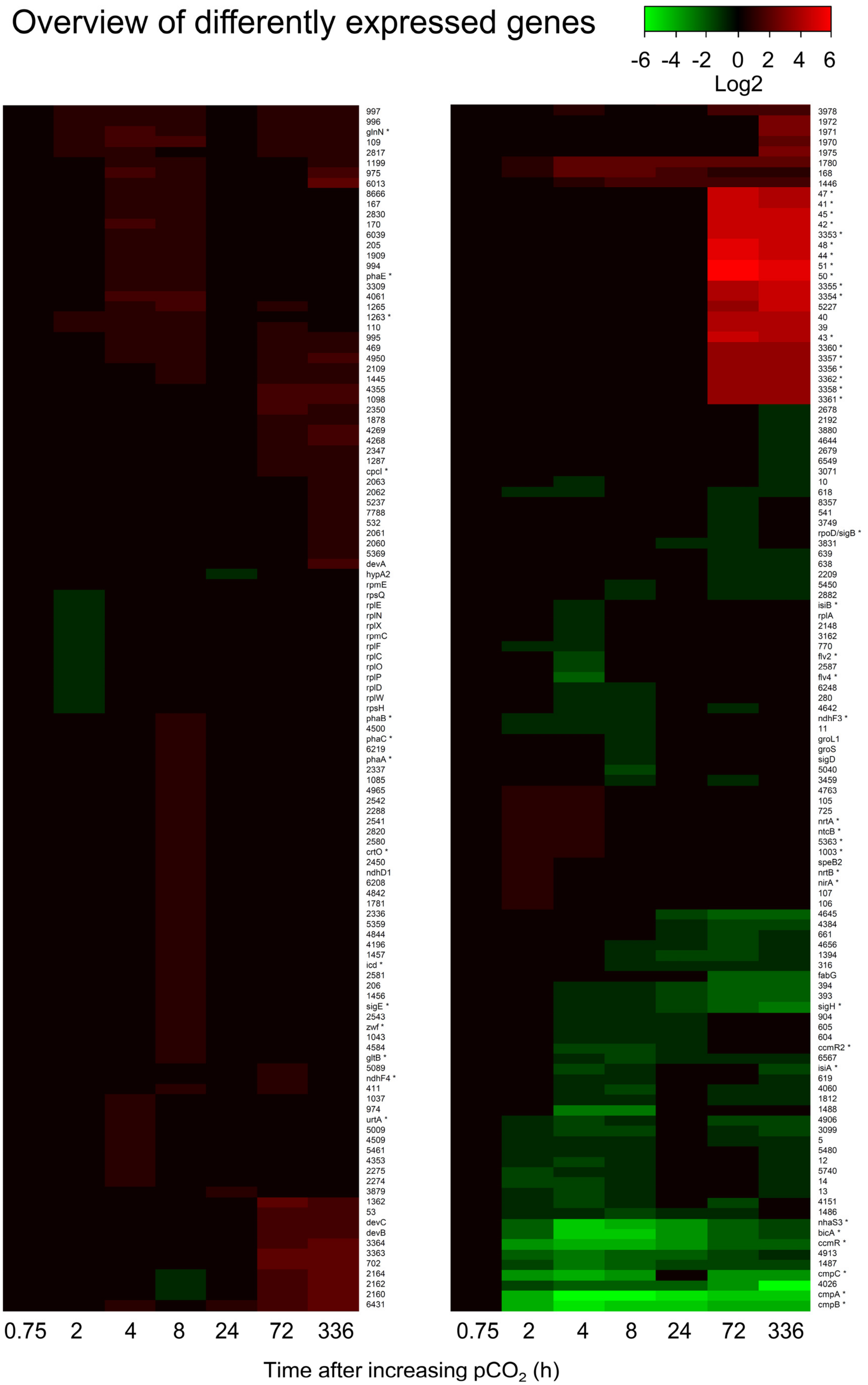
Overview of differently expressed genes
-6 -4 -2 0 2 4 6
Log2
997
996
glnN *
109
2817
1199
975
6013
8666
167
2830
170
6039
205
1909
994
phaE *
3309
4061
1265
1263 *
110
995
469
4950
2109
1445
4355
1098
2350
1878
4269
4268
2347
1287
cpcI *
2063
2062
5237
7788
532
2061
2060
5369
devA
hypA2
rpmE
rpsQ
rplE
rplN
rplX
rpmC
rplF
rplC
rplO
rplP
rplD
rplW
rpsH
phaB *
4500
phaC *
6219
phaA *
2337
1085
4965
2542
2288
2541
2820
2580
crtO *
2450
ndhD1
6208
4842
1781
2336
5359
4844
4196
1457
icd *
2581
206
1456
sigE *
2543
zwf *
1043
4584
gltB *
5089
ndhF4 *
411
1037
974
urtA *
5009
4509
5461
4353
2275
2274
3879
1362
53
devC
devB
3364
3363
702
2164
2162
2160
6431
3978
1972
1971
1970
1975
1780
168
1446
47 *
41 *
45 *
42 *
3353 *
48 *
44 *
51 *
50 *
3355 *
3354 *
5227
40
39
43 *
3360 *
3357 *
3356 *
3362 *
3358 *
3361 *
2678
2192
3880
4644
2679
6549
3071
10
618
8357
541
3749
rpoD/sigB *
3831
639
638
2209
5450
2882
isiB *
rplA
2148
3162
770
flv2 *
2587
flv4 *
6248
280
4642
ndhF3 *
11
groL1
groS
sigD
5040
3459
4763
105
725
nrtA *
ntcB *
5363 *
1003 *
speB2
nrtB *
nirA *
107
106
4645
4384
661
4656
1394
316
fabG
394
393
sigH *
904
605
604
ccmR2 *
6567
isiA *
619
4060
1812
1488
4906
3099
5
5480
12
5740
14
13
4151
1486
nhaS3 *
bicA *
ccmR *
4913
1487
cmpC *
4026
cmpA *
cmpB *
0.75 2 4 8 24 72 336
0.75 2 4 8 24 72 336
Time after increasing pCO2 (h)

Supplementary Figure 4 Overview of all genes responding to rising pCO_2. The expression changes are shown as log2 values. Red indicates significant upregulation and green significant downregulation; non-significant changes are in black. Hierarchical clustering was used to order the genes. Genes with superscript * are also shown in the main text. The complete data set can be found in **Supplementary Table S2**.

2. Supplementary Tables

Supplementary Table 1 Microarray chip loading scheme.

Sample ID	Biological replicate	Chip	Array	Dye	Slide side
t0	1	1	3	Cy5	R
t0	2	1	4	Cy3	R
t0	3	2	11	Cy3	R
t0	4	2	12	Cy5	R
t0.75	1	1	1	Cy5	R
t0.75	2	1	2	Cy3	R
t0.75	3	2	9	Cy3	R
t0.75	4	2	10	Cy5	R
t2	1	1	5	Cy3	L
t2	2	1	4	Cy5	R
t2	3	2	13	Cy5	L
t2	4	2	12	Cy3	R
t4	1	1	3	Cy3	R
t4	2	1	2	Cy5	R
t4	3	2	11	Cy5	R
t4	4	2	10	Cy3	R
t8	1	1	5	Cy5	L
t8	2	1	6	Cy3	L
t8	3	2	13	Cy3	L
t8	4	2	14	Cy5	L
t24	1	1	7	Cy3	L
t24	2	1	6	Cy5	L
t24	3	2	15	Cy5	L
t24	4	2	14	Cy3	L
t72	1	1	7	Cy5	L
t72	2	1	8	Cy3	L
t72	3	2	15	Cy3	L
t72	4	2	16	Cy5	L
t336	1	1	1	Cy3	R
t336	2	1	8	Cy5	L
t336	3	2	9	Cy5	R
t336	4	2	16	Cy3	L

Supplementary Table 2 Complete transcriptome data (see enclosed Excel file).

Supplementary Table 3 *Microcystis* primers used in the RT-qPCR gene expression analysis. The average amplification efficiency (E) ± s.d. of 12-36 samples for each primer pair was calculated using LinRegPCR (version 2012.3).

Primer name	Sequence 5'→ 3' (length)	Gene symbol	Function of complete protein/complex	Locus tag	Accession no. (Genbank)	Expected product size (bp)	Amplification efficiency E	Reference
16SrRNA-F	GTCGAACGGGAATCTTCGGAT (21)	*16S rRNA*	Used here as reference gene for quantification of gene expression	IPF_5548	AM778951.1	132	1.88±0.03	This study
16SrRNA-R	GCTAATCAGACGCAAGCTCTTC (22)							This study
bicA-F	CAAGCTAACGGTCGCATCAT (20)	*bicA*	Low-affinity bicarbonate/sodium symporter	IPF_4911	AM778949.1	157	1.87±0.02	This study
bicA-R	AGGCACATCACTCAAGTCCA (20)							This study
cmpA-F	GTTAAACACCCAGGGTAACGGA (22)	*cmpA*	High-affinity ATP-dependent bicarbonate uptake system	IPF_2181	AM778958.1	180	1.85±0.01	This study
cmpA-R	GCTAACCAGTAACGAATCCAGAAGT (25)							This study
chpX-F	CCTGTCAAGTCCTCCTCTCAT (21)	*chpX*	Low-affinity CO_2 uptake system	IPF_1842	AM778957.1	113	1.89±0.01	This study
chpX-R	TTCAGGATACCCACTACCTCG (21)							This study
chpY-F	ATATCGCCAAAATGCCGACC (20)	*chpY*	High-affinity CO2 uptake system	IPF_1545	AM778958.1	114	1.80±0.02	Sandrini *et al.*, 2014
chpY-R	GACATCATCCGCACCTGTTC (20)							Sandrini *et al.*, 2014
rbcX-F	CGGATCATGACGGTAAGAGAACA (23)	*rbcX*	RuBisCO chaperone, in same operon as genes of small and large subunit of RuBisCO	IPF_2531	AM778933.1	157	1.85±0.02	This study
rbcX-R	ATTCCGATGTCTCTGGTTGACT (22)							This study
ccmM-F	AAGTCCACACCTTCTCTAACCTC (23)	*ccmM*	Carboxysomal protein; in same operon as other carboxysomal genes	IPF_5695	AM778933.1	118	1.87±0.01	Sandrini *et al.*, 2014
ccmM-R	CTGTCGTCGCCAATGTGAA (19)							Sandrini *et al.*, 2014
ccaA-F	ACTCCTGCGGTTAATACTGTGG (22)	*ccaA*	Carboxysomal carbonic anhydrase	IPF_5538	AM778919.1	97	1.87±0.01	Sandrini *et al.*, 2014
ccaA-R	GATAAATGCGATCAGCTTGGGAG (23)							Sandrini *et al.*, 2014
ecaA-F	CCCAAGAACCTTCTCCTGAAATG (23)	*ecaA*	α-type carbonic anhydrase; presumably mainly present in the periplasmic space	IPF_4566	AM778932.1	187	1.86±0.01	Sandrini *et al.*, 2014
ecaA-R	GCCAATTGTTGCAGTTGTTGG (21)							Sandrini *et al.*, 2014
mcyB-F	ATCCCATGCTCAGAGACGTT (20)	*mcyB*	Microcystin synthesis	IPF_375	AM778952.1	163	1.88±0.01	Sandrini *et al.*, 2014
mcyB-R	AGATGTCCGCAGGGATTCAT (20)							Sandrini *et al.*, 2014

Supplementary Table 4 Expression of genes involved in pigment synthesis, photosystems, CCM, C metabolism, C storage, N assimilation, stress response and secondary metabolites (see enclosed Excel file).

References

Sandrini, G., Matthijs, H. C. P., Verspagen, J. M. H., Muyzer, G., and Huisman, J. (2014). Genetic diversity of inorganic carbon uptake systems causes variation in CO_2 response of the cyanobacterium *Microcystis*. *ISME J.* 8, 589–600. doi: 10.1038/ismej.2013.179
